# An Automated Algorithm for Determining Sleep Using Single-Channel Electroencephalography to Detect Delirium: A Prospective Observational Study in Intensive Care Units

**DOI:** 10.3390/healthcare10091776

**Published:** 2022-09-15

**Authors:** Kentaro Matsui, Nobuo Sato, Masafumi Idei, Masako Arakida, Yusuke Seino, Jun-ya Ishikawa, Masashi Nakagawa, Rie Akaho, Katsuji Nishimura, Takeshi Nomura

**Affiliations:** 1 Department of Psychiatry, Tokyo Women’s Medical University, Tokyo 1628666, Japan; 2 Department of Clinical Laboratory, National Center Hospital, National Center of Neurology and Psychiatry, Tokyo 1878551, Japan; 3 Department of Sleep-Wake Disorders, National Institute of Mental Health, National Center of Neurology and Psychiatry, Tokyo 1878551, Japan; 4 Department of Intensive Care Medicine, Tokyo Women’s Medical University, Tokyo 1628666, Japan; 5 Department of Anesthesiology and Intensive Care Medicine, Yokohama City University, Yokohama 2360004, Japan; 6 Department of Nursing, Tokyo Women’s Medical University, Tokyo 1628666, Japan

**Keywords:** intensive care unit, sleep, critically ill patients, delirium, single-channel electroencephalography, under-mattress sleep monitor

## Abstract

The relationship between polysomnography-based objective sleep and delirium in the intensive care unit (ICU) is inconsistent across studies, suggesting limitations in manually determining the sleep stage of critically ill patients. We objectively measured 24-h sleep using a single-channel electroencephalogram (SleepScope [SS]) and an under-mattress sleep monitor (Nemuri SCAN [NSCAN]), both of which have independent algorithms that automatically determine sleep and wakefulness. Eighteen patients (median age, 68 years) admitted to the ICU after valvular surgery or coronary artery bypass grafting were included, and their sleep time was measured one day after extubation. The median total sleep times (TSTs) measured by SS (TST-SS) and NSCAN were 548 (48–1050) and 1024 (462–1257) min, respectively. Two patients with delirium during the 24-h sleep measurement had very short TST-SS of 48 and 125 min, and the percentage of daytime sleep accounted for >80% in both SS and NSCAN. This preliminary case series showed marked sleep deprivation and increased rates of daytime sleeping in ICU patients with delirium. Although data accuracy from under-mattress sleep monitors is contentious, automated algorithmic sleep/wakefulness determination using a single-channel electroencephalogram may be useful in detecting delirium in ICU patients and could even be superior to polysomnography.

## 1. Introduction

Delirium is a prevalent and serious problem in patients admitted to the intensive care unit (ICU). Delirium has been shown to increase the length of ICU stay [1] and worsen subsequent outcomes such as mortality and cognitive functioning [2,3]. Critically ill patients admitted to the ICU are at risk for delirium due to their causative illness, surgical invasion, or inflammation [4]; furthermore, sleep disruption has been noted as a possible cause of delirium onset [5,6,7,8]. Although the interaction between critical illness, delirium, and sleep is complex and their causal relationship is not clear, the importance of sleep disruption in the ICU as a factor associated with delirium has been emphasized in recent guidelines [9]. Sleep disturbances in patients who are critically ill have been caused by the ICU environment, including noise and nursing care, underlying illness, ventilation, pain, and medications; they have also been associated with delirium [10]. Furthermore, circadian rhythm disruption, which is highly correlated with irregular sleep-wake rhythms, has been noted to be associated with delirium [11,12,13].

Hitherto, many of the studies that have examined the association between sleep disturbances and delirium in the ICU have been based on subjective sleep assessments [14,15,16,17,18,19]. Yet, given the potential discrepancy between sleep assessment from the patient’s or nurse’s perspective and objectively recorded sleep measures in the ICU [20,21,22], research to clarify the relationship between objective sleep evaluation and delirium is warranted. Objectively measured sleep fragmentation has been observed in the ICU using polysomnography (PSG), which is the gold standard in sleep evaluation [8,13,21,23,24,25,26,27,28,29,30]. Several studies have already performed PSG in ICU patients who are critically ill. Nevertheless, unlike previous reports showing a link between subjective sleep deprivation and delirium [14,15,17], the relationship between delirium and objectively measured sleep fragmentation is inconsistent across studies [8,13,28,30]. Furthermore, one study indicated that sleep in the ICU was atypical regardless of clinical delirium status; the usual scoring method frequently failed to determine sleep stages [28]. Given the possibility of normalization bias on sleep-determination in the ICU using PSG, sleep staging based on validated algorithms may be even more appropriate for detecting delirium.

Recently, some studies have attempted to objectively assess sleep with single-channel electroencephalography (EEG) [31,32,33]. The device and its algorithms for sleep staging have been validated against PSG [31] and are considered useful for objective measurement of sleep in the ICU. Moreover, another advantage of using single-channel EEG is the capability of non-invasively confirming sleep over a 24-h period [31]. This makes it possible to assess both sleep fragmentation during the night and sleep-wake rhythm disruption due to the propensity to fall asleep during the day. However, no studies have used automatic sleep-scoring algorithms to objectively determine sleep in the ICU and examine the relationship with delirium development.

The objective of this study was to clarify the utility of single-channel EEG and its automatic sleep-scoring algorithms for detecting delirium. We set out to conduct a 24-h objective sleep assessment using SleepScope (SS), a single-channel EEG, in patients admitted to the ICU. We also used the Nemuri SCAN (NSCAN), a recently developed under-mattress sleep monitor [34,35], to assess sleep and wakefulness. We hypothesized that both sleep deprivation at night and more daytime sleep from the single-channel EEG and under-mattress sleep monitor are associated with the development of delirium. As a secondary endpoint, we examined the reproducibility on the validity of sleep-wake assessments from under-mattress sleep monitors. Additionally, we investigated the relationship between these objective sleep measures and subjective nighttime sleep assessments.

## 2. Materials and Methods

### 2.1. Study Setting and Sample

This prospective observational study was approved by the Institutional Review Board of Tokyo Women’s Medical University (no. 5690). The study was conducted from February 2021 to March 2021 in two general ICUs (32 beds in total) at Tokyo Women’s Medical University Hospital (a 1200-bed tertiary teaching hospital in Tokyo, Japan). ICU staff primarily treated perioperative patients with serious complications (especially those undergoing cardiac surgery) and hospitalized patients with acute respiratory failure, septic shock, and other serious illnesses requiring ventilation, continuous renal replacement therapy, and extracorporeal support. The ratio of registered nurses to patients was 1:2 during the study period. The ICU was set to have low intensity lighting between 10:00 p.m. and 6:00 a.m. At least twice a day, nurses checked the patients’ disorientation, including names and dates.

Patients aged >20 years who had undergone coronary artery bypass grafting or cardiac valve surgery and who were subsequently admitted to the ICU for at least 72-h were eligible for a 24-h sleep evaluation using the SleepScope (SS), the Nemuri SCAN (NSCAN), and a questionnaire. Patients with brain dysfunction, psychiatric disorders, dementia, alcohol or drug abuse, and post-cardiopulmonary arrest were excluded. Informed consent and written confirmation were obtained from all patients prior to participation.

### 2.2. Data Collection

Demographic and clinical data including age, sex, body mass index, type of surgery and operation time, anesthesia time, period of artificial respiration, medications used, length of ICU stay, and length of hospitalization were collected from the patients’ electronic medical records. We calculated Acute Physiology and Chronic Health Evaluation (APACHE) II scores at admission to the ICU. Cognitive function was evaluated by either N.S. or M.A. using the Mini-Mental State Examination (MMSE) after consent was obtained. The MMSE is widely used to quantify cognitive function [36], and a score of 23 or below was considered to indicate dementia [37].

In our hospital, nurses routinely used the Intensive Care Delirium Screening Checklist (ICDSC) in the ICU and the Delirium Rating Scale-Revised-98 (DRS-R-98) in the general ward. The ICDSC is a tool to assess delirium in the ICU, and a score of 4 or higher was considered delirium [38,39]. The DRS-R-98 is a tool to assess delirium in hospital wards, and a score of 10 or higher was considered delirium [40,41]. The ICDSC was administered twice a day during the ICU stay, and the DRS-R-98 was administered in the general ward for at least five days.

### 2.3. SleepScope Analysis

The SS is a single-channel portable EEG from SleepWell (Osaka, Japan). SS recordings were normally made from 12:00 p.m. on the day following extubation to 12:00 p.m. the day thereafter. However, we allowed for start time variations due to diet or other care, or upon patient request. Even when the start of recording was advanced or delayed, measurements were performed for 24 h from the start time. Both SS recording and analysis methods are described in detail elsewhere [31]. Briefly, one SS electrode was placed at the center of the forehead and the other at the left mastoid process. The data obtained with SS were transferred to a cloud service (SEAS-G, SleepWell, Osaka, Japan), where spectral analysis of the EEG data was performed every 30-s epoch. The data were analyzed in five stages: wakefulness, stage 1 (N1), stage 2 (N2), slow wave sleep (SWS), and rapid eye movement sleep (REM). Stage information was provided along with time stamps, and EEG traces were available for download. These timing data allowed the SS results to be synchronized with other datasets used in this study. This service was approved for medical device certification (225ADBZX00020000) in Japan. Since the sleep stage of SS was validated by summing N1 and N2 in comparison with PSG [31], we also combined N1 and N2 in this study. The sum of N1 + N2, SWS, and REM was used as total sleep time (TST) measured by SS (TST-SS). Each sleep stage and TST-SS was extracted from 24-h and nighttime recordings (8-h from 22:00 to 6:00), respectively.

### 2.4. Nemuri SCAN Measurement

The NSCAN (Paramount Bed Corporation, Tokyo, Japan) is a non-wearable sleep monitor that is placed under the mattress and automatically identifies sleep-wake cycles by assessing body movement, breathing, and heart rate activity, which has been validated in healthy individuals [34]. The NSCAN was placed under the top half of each mattress prior to patient admission and remained in place until discharge from the ICU. The NSCAN data used in this study were limited; we used only data from the day after extubation and the 24-h measurements using SS for the same periods.

NSCAN has a highly sensitive pressure sensor that detects movement over the mattress, identifies sleep onset and wake-up, and calculates an activity score every minute. This score reflects the intensity and frequency of body movements, excluding small movements due to breathing or heartbeat. NSCAN uses a proprietary “sleep-wake” algorithm to automatically detect sleep and wake states on a minute-by-minute basis and calculate sleep variables [34]. For example, if the NSCAN recorded 60-s of sleep at 9:30 p.m., two 30-s sleep epochs were recorded from 9:30 p.m. and 9:30:30 p.m. The total time of epochs judged as sleep was used as total sleep time measured by NSCAN (TST-N).

### 2.5. Sleep Questionnaire

At the time of obtaining research consent, a self-administered questionnaire, the Insomnia Severity Index (ISI) [42,43], was used to ascertain insomnia severity prior to the surgery. The ISI includes seven items: severity of difficulty falling asleep and maintaining sleep, satisfaction with current sleep patterns, interference with daily functioning, appearance of impairment attributed to the sleep problem, and level of concern about insomnia. Each item is scored on a 5-point Likert scale (0 = not at all, 4 = extremely). A cutoff score of 10 was considered optimal for detecting insomnia [44].

Subjective sleep quality and sleep duration during the night were assessed. To assess subjective sleep quality, we used the items used in the Sleep Heart Health Study [45,46,47]. A 5-point Likert-type questionnaire, in which higher scores indicate higher sleep quality, was used to evaluate depth of sleep (light to deep) and feeling of restfulness from sleep (restless to restful) the previous night. Subjective sleep duration in the previous night was also obtained from the patients. These subjective assessments were performed at 10 am the next morning during the 24-h measurement of SS and NSCAN.

### 2.6. Statistical Analyses

The median and range (i.e., the minimum and maximum values) were used to summarize nominal variables. For the statistical analysis, we excluded patients from the study if the SS could not be measured for any reason. The agreement rate, sensitivity, and specificity were calculated based on SS and NSCAN data measured at the same time; according to the method of Nagatomo et al. [35], the NSCAN data indicating sleep or wake were validated by the SS results. The agreement rate represented the percentage of epochs with the same judgment, that is, the percentage of epochs per total epoch in which both NSCAN and SS judged sleep or wakefulness. Sensitivity was the ratio of the number of epochs judged as sleep by NSCAN to the number of epochs judged as sleep by SS. Specificity represented the ratio of the number of epochs judged as awake by NSCAN to the number of epochs judged as awake by SS. If the SS could not determine sleep or wakefulness due to electrode disconnection or if the NSCAN record indicated either “out of bed” or “battery disconnected” status, both SS and NSCAN data at that time were excluded from the analysis. We then verified the objective sleep parameters from SS (TST-SS, N1 + N2, SWS, and REM) and NSCAN at night to identify their subjective sleep quality correlation (depth and restfulness) and subjective sleep duration at night; we employed Spearman’s rank correlation coefficient, as it is preferable for small sample sizes and is less prone to artifacts due to outliers. SPSS statistics version 26 (SPSS Japan, Inc., Tokyo, Japan) was used for statistical analysis. *p* values < 0.05 were considered statistically significant.

## 3. Results

Twenty patients who were admitted to the ICU during the study period met the inclusion criteria. Of these, one patient who refused to undergo 24-h sleep measurement and one patient whose SS could not be measured due to battery failure were excluded. Finally, 18 patients (11 male and seven female) were included in the study. Descriptive data for all patients from the delirium and non-delirium groups are presented in Table 1.

The median start time of SS measurements was 12:51 (11:46–16:11). SS was performed for 24-h in all cases. NSCAN results at the same time were used for analysis, except for two cases where the NSCAN could not be placed. Using 30-s as one epoch, 1714 epochs from the SS were determined to be errors and difficult to distinguish between sleep and wakefulness. There were 735 epochs of “out of bed” from the NSCAN, with no episodes of “battery disconnected.” Finally, 26,576 epochs during the day and 14,551 epochs at night (22:00_05:59), totaling 41,127 epochs, were used to check SS and NSCAN concordance rates. Based on SS and NSCAN measurements over the same period, the agreement, sensitivity, and specificity (95% confidence intervals) for determining sleep or wakefulness during the day are presented in Table 2.

The nighttime sleep parameters and results obtained from SS, NSCAN, and the questionnaire are shown in Table 3. In one non-delirious case, most of the SS data at night were in error due to electrode displacement. This case is excluded in the following data. Except for two cases (one delirious and one non-delirious), 8-h of NSCAN were fully recorded. In two cases in which sleep was measured under delirious state (nos. 1 and 2), TST-SS at night was less than 30 min. REM deprivation at night was also observed in these cases, as well as in seven of the 16 cases examined in the non-delirium state.

Correlations with objective sleep measurements at night (TST-SS at night, N1 + N2, SWS, REM, and TST-N at night) and subjective sleep quality (depth and restfulness) or subjective sleep duration at night are shown in Table 4. Deeper sleep quality was significantly correlated with more TST-SS at night (r = 0.519, *p* = 0.039), more N1 + N2 (r = 0.540, *p* = 0.031), and more REM (r = 0.559, *p* = 0.024), but not with SWS (r = −0.218, *p* = 0.417) and TST-N at night (r = 0.286, *p* = 0.322). More restful sleep was significantly correlated with more TST-SS at night (r = 0.535, *p* = 0.033) and more N1 + N2 (r = 0.533, *p* = 0.033), but not with SWS (r = 0.265, *p* = 0.321), REM (r = 0.400, *p* = 0.124), and TST-N at night (r = 0.026, *p* = 0.931). Longer sleep duration was significantly correlated with more TST-SS at night (r = 0.638, *p* = 0.035) and more N1 + N2 (r = 0.638, *p* = 0.035), but not with SWS (r = 0.050, *p* = 0.884), REM (r = 0.355, *p* = 0.284), and TST-N at night (r = 0.585, *p* = 0.075).

**Figure 1:**
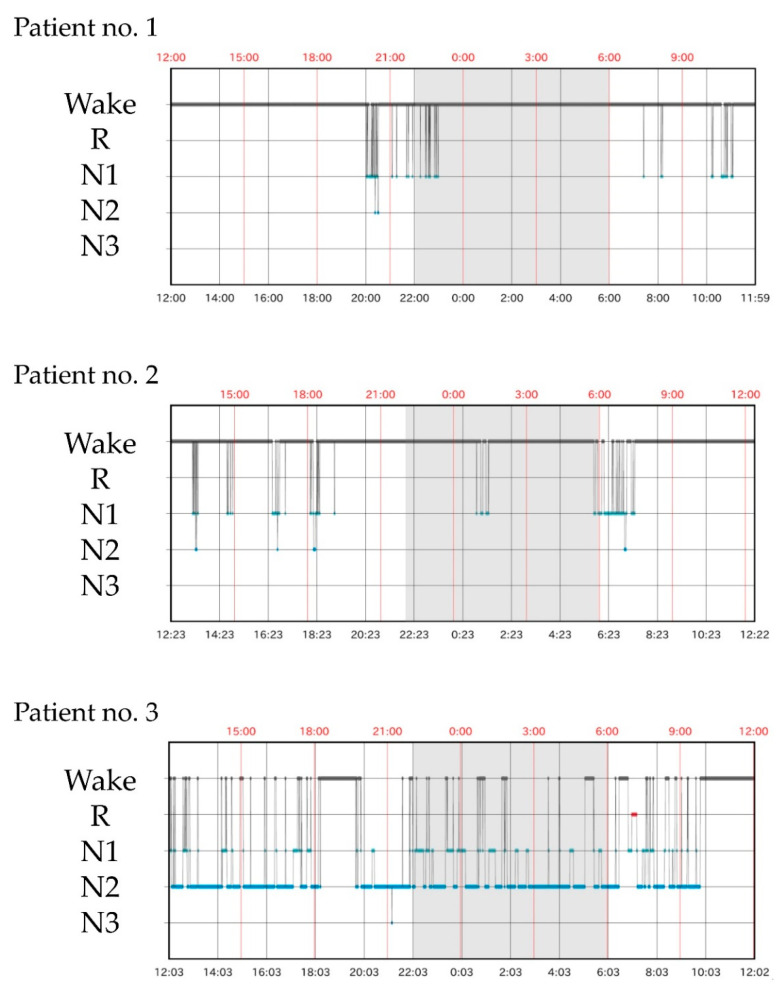
Twenty-four h sleep architecture measured with SS. Patients 1 and 2 had delirium during sleep measurements and showed extreme sleep deprivation throughout the day and night. In patient 3, sleep measurements were performed during recovery from delirium and showed markedly prolonged sleep duration and a total sleep time of 17.5-h.

Delirium severity scores before and during the 24-h sleep measurement, TST and daytime sleep time obtained from SS and NSCAN, and the percentage of daytime sleep time and medications used during the sleep study are presented in Table 5. Delirium occurred in three patients during ICU admission. Patients 1 and 2 had delirium before and during the sleep measurements. Patient 3 experienced delirium before the initiation of sleep measurements, recovered by the evening and was free from delirium the next morning. Regarding concomitant medications, dexmedetomidine was used in 10 patients, opioids in six patients (all fentanyl), and catecholamine in 15 patients (dopamine hydrochloride for 12, dobutamine hydrochloride for seven and noradrenaline for three). None of the patients received γ-aminobutyric acid-A agonists, including midazolam or propofol. Patient 1, who presented with delirium, received antipsychotics (haloperidol) for agitation. As shown in Figure 1, TST-SS in patients 1 and 2 was extremely short (48 and 125 min, respectively). TST-N in patient 2 tended to be shorter (462 min). Except for NSCAN data missing in patient 1, the percentage of daytime sleep in patients 1 and 2 accounted for more than 80% of both SS and NSCAN. In contrast, in patients without delirium during the 24-h measurement, the median percentage of daytime sleep accounted for 45.5 (0–70.8)% of SS results and 58.7 (51.6–72.8)% of NSCAN results, all below 80%. Patient 3, who had delirium before but not during the 24-h sleep measurement, had the longest TST-SS during the sleep study (see Figure 1). TST-N was also longest in patient 3, but similar lengths of TST-N were also observed in several other cases.

## 4. Discussion

This is the first study to assess 24-h sleep using single-channel EEG and under-mattress sleep monitors, and their automated sleep-determination algorithms in ICU patients, to examine its association with delirium. In two cases in which a 24-h sleep study was performed during a delirium episode, both patients had extremely short nighttime sleep, with daytime sleep accounting for >80% of total sleep. These results indicated that nocturnal sleep deprivation and the disruption of sleep-wake rhythm are associated with delirium, which is consistent with our hypothesis.

Previous studies using PSG in the ICU have emphasized REM deprivation in patients with delirium [8,13,30]. Although REM deprivation in the two patients with delirium also occurred in this study, the uniqueness of REM deprivation in patients with delirium was not noticeable, since REM deprivation occurred in approximately 40% of the patients without delirium in. Instead, of note, pronounced nocturnal sleep deprivation was observed in patients with delirium in the present study. Some studies have reported that TST at night tends to be short in patients with delirium [13,30], and another study indicated that TST at night was comparable regardless of delirium status [48]. The reason for the difference between these previous studies and this study is unclear. Given that EEGs in delirium cases could be nonspecific, making it difficult to classify sleep stages [28], it is possible that in the current analysis based on the SS algorithm, all of the nonspecific EEGs that could have occurred in patients with delirium were scored as awake. This can be seen as a vulnerability, but also as a potential strength. Sleep stages measured using PSG can vary among the technologists analyzed [49,50]; moreover, in the ICU setting, reliability in scoring sleep stages can be poor even in non-delirious cases [23,28]. Therefore, a simplified method of determining sleep stages based on a specific uniformed algorithm may have an advantage when detecting delirium. The 24-h sleep measurement in the ICU using single-channel EEG may be applicable not only to post-operative patients but also to those with acute infections, metabolic disturbances, and respiratory diseases. It may also be useful for the risk assessment of delirium in general wards or dementia care facilities. Nevertheless, due to the small number of cases, pronounced nocturnal sleep deprivation during delirium observed in this study warrants a follow-up investigation. Conducting further studies in various settings and with a larger number of patients is imperative before dissemination into clinical practice.

The delirium cases in this study compensated for much of their sleep with daytime sleeping, albeit for short intervals. Thus, a high percentage of daytime sleep may also be a useful delirium-related factor. A study examining the relationship between 24-h sleep-wake rhythms, circadian rhythms, and delirium found a loss of melatonin/cortisol circadian rhythms in patients with delirium, while finding no difference in daytime sleep between patients with and without delirium [13]. Some studies using actigraphy, which utilizes acceleration sensors to estimate sleep [51], have reported impaired rest-activity in patients with ICU delirium [52,53,54], while others have reported scant differences in rest-activity between patients with and without delirium [55]. In this study, data on patient 2 suggest a higher percentage of daytime sleep both in SS and NSCAN. The under-mattress sleep monitor, as well as actigraphy, may be useful only when assessing the delirium risk through the evaluation of sleep-wake rhythms. Meanwhile, a trend toward shortened sleep duration at night and prolonged daytime sleeping were observed even in patients who did not develop delirium. Sleep fragmentation at night [21,24,25,26,27,29,56] and prolonged daytime napping [27,56] has already been reported to occur in the ICU setting; thus, sleep-wake rhythm disruption at some level may have little pathological significance on delirium development.

This study also examined the concordance rate between SS and NSCAN, which was almost identical to the validation results of the PSG and NSCAN, characterized by a high sensitivity and specificity of approximately 40% [35]. The over-estimation of sleep in the ICU environment by the under-mattress sleep monitor was similar to the results obtained when sleep was measured using actigraphy [21,57]. As with actigraphy, under-mattress sleep monitors may not provide accurate sleep measurement in the ICU. Future research should examine the utility of a better algorithm for analyzing sleep with wrist actigraphy [58] or multi-sensor wearables consisting of accelerometers and pulse rates for sleep assessment [59,60] in the ICU setting.

The relationship between the results of single-channel EEG or an under-mattress sleep monitor and subjective sleep assessment is another important finding of this study. Subjective sleep quality (depth and restfulness) and sleep duration showed a moderate association with TST-SS. Depth of sleep was also associated with REM. Thus far, the most commonly used subjective measure of sleep in the ICU is the Richards-Campbell Sleep Questionnaire [27,35,61,62,63]. The correlation between PSG measurements and Richards-Campbell Sleep Questionnaire scores was significant but limited; the correlation was reported to be moderate [61], which is identical to those between TST-SS and subjective sleep measures in the present study. Given the simplicity of the scoring, the subjective sleep questionnaires used in this study (sleep depth, restfulness, and subjective sleep duration) [45,46,47] may also be useful in future studies. In contrast, TST obtained with the under-mattress sleep monitor in the present study showed only a nonsignificant trend of correlation with subjective sleep duration and a negligible relationship with sleep quality assessments. This result is similar to previous studies comparing actigraphy with subjective sleep ratings [64]. In the present study, subjective nighttime sleep quality was impaired regardless of delirium status, which is consistent with previous studies [27,35,61,62,63]. Likewise, we did not find an association between delirium and insomnia prior to ICU admission in this study, which is inconsistent with a previous study [65]. Based on these findings, the relationship between subjective sleep ratings and delirium may be limited in the ICU.

TST-SS in patient 3 showed markedly prolonged sleep duration. In this case, delirium occurred in the morning before sleep measurements began, but then disappeared in the evening, and no delirium was observed thereafter. Thus, this can be a valuable finding regarding 24-h of sleep during recovery from delirium, which was observed coincidentally. In general, humans require restorative sleep with prolonged sleep duration after sleep deprivation [66,67]. Since almost complete sleep deprivation occurred during delirium in the setting of this study, as with patients 1 and 2, it is possible that compensatory prolonged sleep occurred during recovery in the ICU. It is also interesting to note that patient 3 had poor subjective nighttime sleep quality despite long hours of sleep. However, to date, no study has presented detailed sleep measurements at the time of improved delirium in the ICU. Short-term sleep deprivation and accumulation of sleep debt may well contribute to the mechanism of delirium, since various medications with sleep stabilizing effects, such as melatonin, melatonin receptor agonists, orexin antagonists, and dexmedetomidine, have been noted to prevent the onset of delirium [68,69,70]. Whether prolonged sleep always occurs during the recovery phase of delirium is not clear, and future investigations are warranted.

This study has some limitations. First, and most important, the SS algorithm used in the analysis was not optimized for the ICU. In addition, sleep stages N1 and N2 in the SS were not validated individually, as their sum was validated against the PSG stage determination. Subjective sleep ratings were also not validated in the ICU setting. Second, sleep measurements in this study were conducted the day after extubation. The administration of various agents including dexmedetomidine and catecholamines might have affected patients’ sleep. Immobility due to surgical invasion and connection to therapeutic cables, tubes, and cords may also have compromised the results, especially for NSCAN. These are challenges that are difficult to avoid when measuring the sleep of ICU patients at risk of delirium. Third, the sample size of this study was small; we could only present cases that exhibited specific characteristics in testing our hypotheses. Yet, many studies that have examined sleep and delirium in the ICU have involved similarly small sample sizes [8,28,29,30,48,52]. In addition to the small sample size, there were some missing values. Since the under-mattress sleep monitor was not used in patient 1, it was impossible to fully assess the association between sleep-wake rhythm disruption and delirium in this case. Furthermore, many patients indicated that they did not know their own sleep duration, which possibly contributed to the lower statistical power in the correlation analysis. Fourth, only a small number of patients experienced delirium; the rate of delirium development was clearly lower than in previous studies [8,13,29,30,48]. Therefore, we could not employ a group comparison between the delirium and non-delirium groups. One possible reason for the low number of delirium patients may be that we limited the target population to scheduled surgery patients and did not include patients who require emergency surgery. Finally, we did not use raw EEG data obtained with SS. Since the raw EEG data may be useful in detecting delirium [71,72], we plan to study this in the future.

## 5. Conclusions

In this study, we evaluated 24-h sleep using a single-channel EEG and an under-mattress sleep monitor in patients admitted to the ICU after valve and bypass surgery. Sleep deprivation at night and disruption of sleep-wake rhythm were observed in two patients who presented with delirium. A marked prolongation of sleep duration in another case may have occurred as a restorative reaction against delirium. This study demonstrates the potential usefulness of single-channel EEG and its automated algorithms for determining sleep for investigating delirium. Findings from this study may contribute to the feasibility of ICU sleep research and may lay the foundation for future clinical trials for the early detection and intervention of delirium. As this is a preliminary report based on a small number of cases, the results of this study should be followed up in future studies with larger sample sizes.

## Figures and Tables

**Table 1:** Demographic variables, preoperative and intraoperative status, and hospitalization-related variables.

	All Patients (*n* = 18)	Delirium (*n* = 3)	Non-Delirium (*n* = 15)
Age, median [range], year	68 [32–81]	67 [57–76]	68 [32–81]
Male, *n* (%)	11 (61.1%)	3 (100%)	8 (53.3%)
BMI, median [range], kg/m^2^	21.4 [17–26.7]	19 [18.7–21.3]	21.5 [17–26.7]
MMSE, median [range], point	29 [24–30]	28 [24–30]	30 [25–30]
ISI, median [range], point	7.5 [1–17]	6 [5–7]	9 [1–17]
**Surgery**			
Coronary artery bypass grafting, *n* (%)	7 (38.9%)	1 (33.3%)	6 (40.0%)
Valvular surgery, *n* (%)	11 (61.1%)	2 (66.7%)	9 (60.0%)
Anesthesia time, median [range], h: min	7:05 [4:15–9:34]	6:59 [4:22–8:42]	7:11 [4:15–9:34]
Operation time, median [range], h: min	5:57 [3:11–8:13]	5:46 [5:41–7:02]	6:05 [3:11–8:13]
APACHE II, median [range], min	18 [6–26]	25 [14–26]	18 [6–22]
Artificial respiration period, median [range], day	1 [0–2]	1 [1–1]	1 [0–2]
Length of ICU stay, median [range], day	4 [3–14]	4 [4–14]	4 [3–10]
Length of hospitalization, median [range], day	29.5 [17–71]	34 [22–43]	28 [17–71]

BMI, body mass index; MMSE, Mini-Mental State Examination; ISI, Insomnia Severity Index; APACHE II, Acute Physiology and Chronic Health Evaluation II; ICU, intensive care unit.

**Table 2:** Validity of Nemuri SCAN (NSCAN) with SleepScope (SS) (*n* = 16).

	Daytime		Nighttime	
		95% CI		95% CI
Agreement	0.563	0.559–0.568	0.713	0.705–0.720
Sensitivity	0.929	0.921–0.936	0.944	0.937–0.949
Specificity	0.403	0.400–0.406	0.388	0.379–0.396
PPV	0.405	0.402–0.408	0.685	0.680–0.689
NPV	0.928	0.921–0.936	0.830	0.811–0.847
PLR	1.556	1.535–1.577	1.541	1.509–1.572
NLR	0.176	0.157–0.197	0.145	0.128–0.165

Both NSCAN and SS were measured for 24-h, of which results from 10:00 p.m. to 5:59 a.m. are shown as nighttime and others as daytime. CI, confidence interval; NLR, negative likelihood ratio; NPV, negative predictive value; PLR, positive likelihood ratio; PPV, positive predictive value.

**Table 3:** Results of nighttime sleep measurements and subjective sleep assessment.

	Delirium (*n* = 3)			Non-Delirium(*n* = 14) ^1^
	Patient 1	Patient 2	Patient 3	
**SS at night**				
TST-SS at night, min (%TIB)	10.5 (2.2)	19.5 (4.1)	420 (87.5)	310.3 [59–397.5]
N1 + N2 at night, min (%TST-SS)	10.5 (100)	19.5 (100)	420 (100)	310.3 [59–379.5]
N3 at night, min (%TST-SS)	0 (0)	0 (0)	0 (0)	0 [0–7.5]
REM at night, min (%TST-SS)	0 (0)	0 (0)	0 (0)	5.3 [0–54]
**NSCAN at night**				
TST-N at night, min	—	31	436	412 [294–472]
**Subjective sleep assessments at night**				
Quality of sleep: light/deep	1	1	1	2 [1–5]
Quality of sleep: restless/restful	1	1	1	2 [1–4]
Sleep duration, min	0	Not sure	210	180 [60–390]

^1^ Results after excluding one case in which most of the nighttime SS measurement results were in error. Data are presented as median [range]. “—” indicates missing data. Subjective sleep quality was evaluated on a scale from 1 (light or restless) to 5 (deep or restful). TIB, time in bed; TST-SS, total sleep time measured by SleepScope; TST-N, total sleep time measured by NSCAN; REM, rapid eye movement sleep.

**Table 4:** Correlation between nighttime sleep measurements (SS/NSCAN) and subjective sleep assessments.

	Subjective Sleep Assessments		
	Quality of Sleep: Light/Deep	Quality of Sleep: Restless/Restful	Sleep Duration
**SS at night**			
TST-SS	0.519 *	0.535 *	0.638 *
N1 + N2	0.540 *	0.533 *	0.638 *
SWS	−0.218	0.265	0.050
REM	0.559 *	0.400	0.355
**NSCAN at night**			
TST-N	0.286	0.026	0.585

Spearman’s rank correlation coefficient was used for the analysis. Patients with missing subjective sleep assessments were excluded from the analysis. One of the patients, most of whose nighttime measurements were in error, was also excluded from the analysis (see Table 3). TST-SS, total sleep time measured by SS; SWS, slow wave sleep; REM, rapid eye movement sleep; TST-N, total sleep time measured by NSCAN. * *p* < 0.05.

**Table 5:** Delirium scores, sleep measures, and medications.

	Delirium (*n* = 3)			Non-Delirium (*n* = 14) ^1^
	Patient 1	Patient 2	Patient 3	
**Delirium scores**				
ICDSC score (day at the test, AM)	5 ^2^	7 ^2^	6 ^2^	0 [0–2]
ICDSC score (day at the test, PM)	5 ^2^	8 ^2^	3	0 [0–2]
ICDSC score (next day, AM)	2	7 ^2^	NA	1 [0–1]
DRS-R-98 score (next day, AM)	NA	NA	6	0 [0–0]
**SS variables**				
Total measurement time, min	1409	1440	1440	1440 [931–1440]
Total error time, min	31	0	0	0 [0–509]
TST-SS, min	48	125	1050	548 [136.5–872.5]
Daytime sleep duration, min	37.5	105.5	630	225 [51.5–475]
Ratio of daytime sleep, %	80.7	84.4	60.0	45.5 [0–70.8]
**NSCAN variables**				
Total measurement time, min	—	1440	1440	1429 [1249–1440]
Total error time, min	—	0	0	11 [0–191]
TST-N, min	—	462	1257	1036 [614–1249]
Daytime sleep duration, min	—	431	821	592.3 [320–805]
Ratio of daytime sleep, %	—	93.3	65.3	58.7 [51.6–72.8]
**Medications**				
Dexmedetomidine	Y	Y	Y	6 (40%)
Opioids	N	Y	N	5 (33.3%)
Catecholamine	Y	Y	Y	12 (80%)
Antipsychotics	Y	N	N	0 (0%)

^1^ Data are presented as median [range] or number (percentage). ^2^ Higher than the cutoff for delirium (ICDSC ≥ 4 or DRS-R-98 ≥ 10). Data are presented as median [range]. “—” indicates missing data. TST-SS, total sleep time measured by SS; TST-N, total sleep time measured by NSCAN; ICDSC, Intensive Care Delirium Screening Checklist; DRS-R-98, Delirium Rating Scale-Revised-98; M, male; F, female; NA, not assessed; Y, yes; N, no.

## Data Availability

The data that support the findings of this study are available on request from the corresponding author. The data are not publicly available due to privacy or ethical restrictions.
